# Supplementary material for: Identification of a Multi-Messenger RNA Signature as Type 2 Diabetes Mellitus Candidate Genes Involved in Crosstalk between Inflammation and Insulin Resistance
Source: Biomolecules. 2022 Sep 2;12(9):1230. doi: 10.3390/biom12091230 (PMC9496026; doi:10.3390/biom12091230)
Supplement: Supplementary file 1 [file biomolecules-12-01230-s001.zip › supplementary figures.pdf]

Identification of a Multi–Messenger RNA Signature As Type 2 diabetes mellitus Candidate genes involved in crosstalk between inflammation and insulin resistance

Running title: mRNAs in type 2 diabetes mellitus

Hebatalla Said Ali* [1], Mariam Sameh Boshra* [1], Sara H.A. Agwa[2], Mohamed S. Abdel Hakeem [3], Mahmoud Shawky El Meteini[4] and Marwa Matboli* [1]

[1] Medical Biochemistry and Molecular biology department, Faculty of Medicine, Ain Shams University, Abbassia, Cairo, Egypt, P.O. box 11381
[2] Clinical Pathology, Medical Ain shams research institute, Ain shams university, Abbassia, Cairo, Egypt, P.O. box 11381
[3] Institute of Immunology, University of Pennsylvania, Philadelphia, United States
[4] Department of General Surgery, the school of Medicine, University of Ain Shams, Cairo 11591, Egypt
* Correspondence: hebatallahsaid@med.asu.edu.eg, mariamsameh09@med.asu.edu.eg ; drmarwa_matboly@med.asu.edu.eg (M.M.)

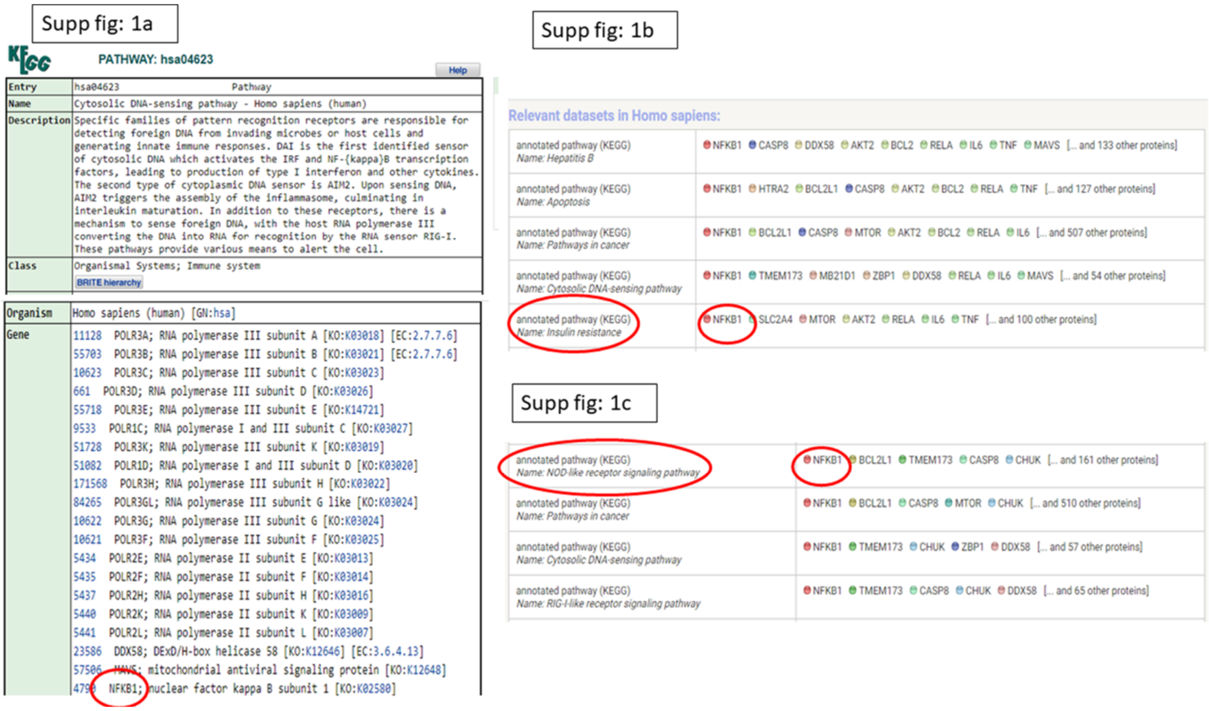

Figure S1. Showing the retrieval of NFKB1 as an important effector in the pathway of type 2 DM development. Supp. fig.: 1a, using KEGG pathway database, NFKB1 is related to STING pathway. Supp. fig.: 1b, shows the involvement of NFKB1 in insulin resistance using KEGG pathway. Supp. fig.: 1c, shows its relation with NOD like receptor pathway using KEGG pathway data base.

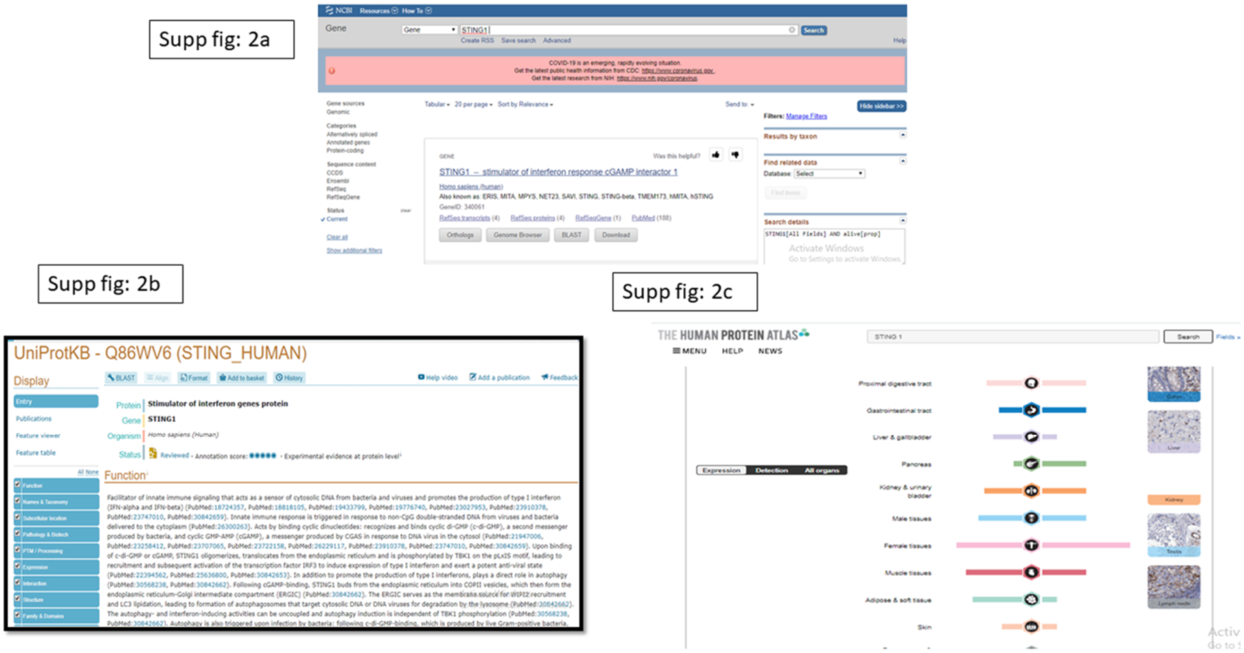

Figure S2. Showing the retrieval of STING1 (TMEM173) as an important intermediate in cGAS/CGAMP/STING pathway. Supp. fig.: 2a, retrieval of STING1 (TMEM173) from NCBI. Supp. fig.: 2b, shows the different functions of STING1 using UNIPROT data base. Supp. fig.: 2c, shows its expression in different tissues using Human Protein Atlas data base.

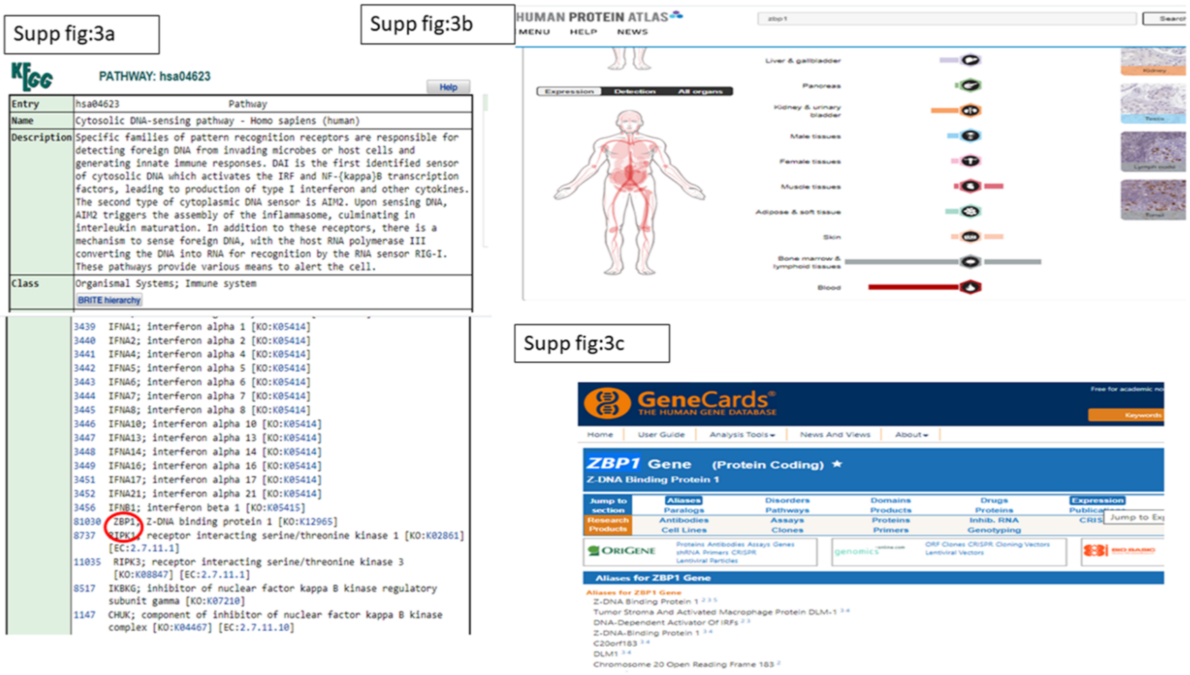

Figure S3. Showing the retrieval of ZBP1 as an important intermediate in cGAS/CGAMP/STING pathway. Supp. fig.: 3a, Shows the relation of ZBP1 to STING pathway by using KEGG pathway data base. Supp. fig.: 3b, shows the different tissue expression of ZBP1 by Human Protein Atlas data base. Supp. fig.: 3c, shows its retrieval from gene cards data base.

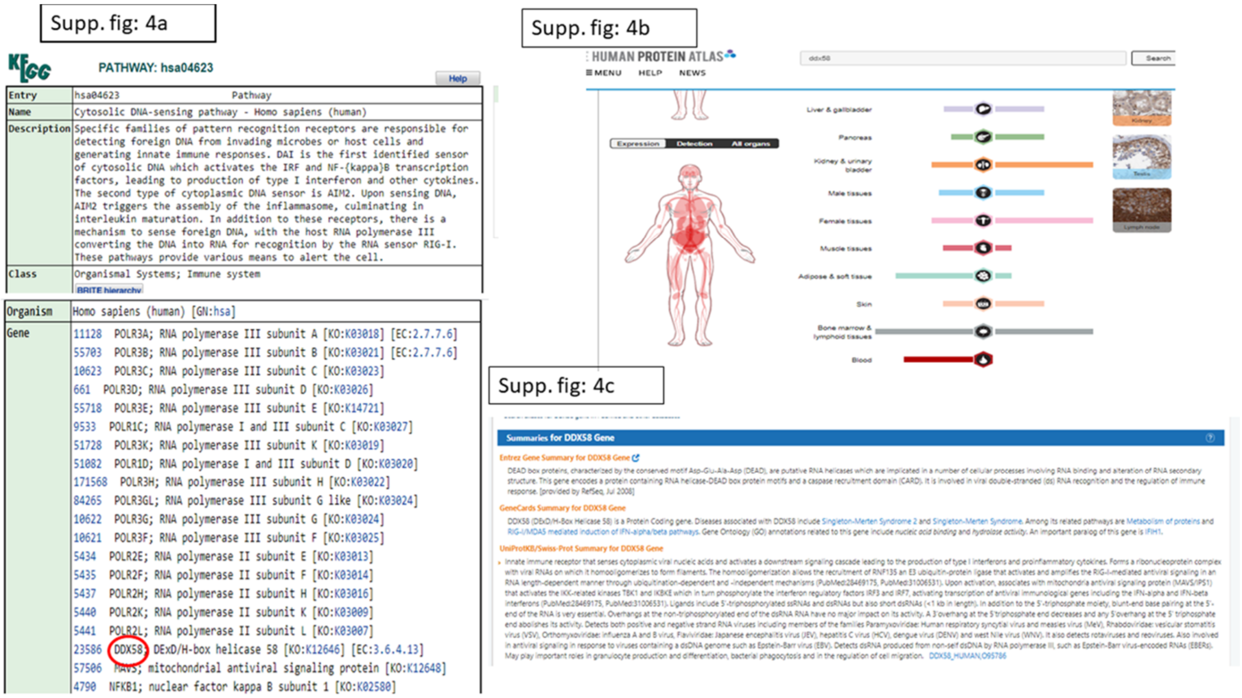

Figure S4. Showing the retrieval of DDX58 as an important intermediate in cGAS/CGAMP/STING pathway. Supp. fig.: 4a, Shows the relation of DDX58 to STING pathway by using KEGG pathway data base. Supp. fig.: 4b, shows the different tissue

expression of DDX58 by Human Protein Atlas data base. Supp. fig.: 4c, shows its retrieval from gene cards data base.

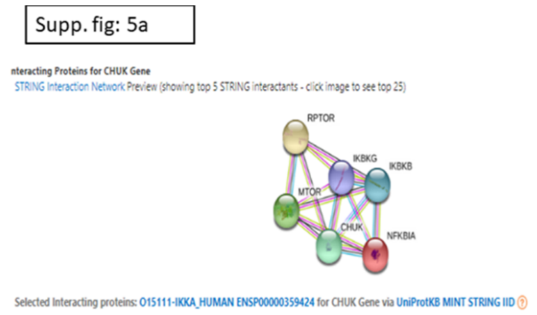

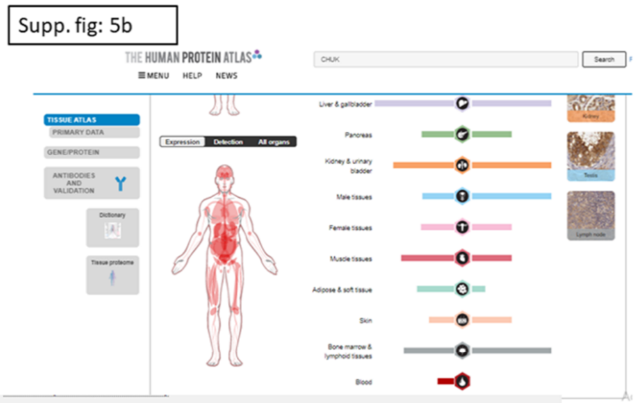

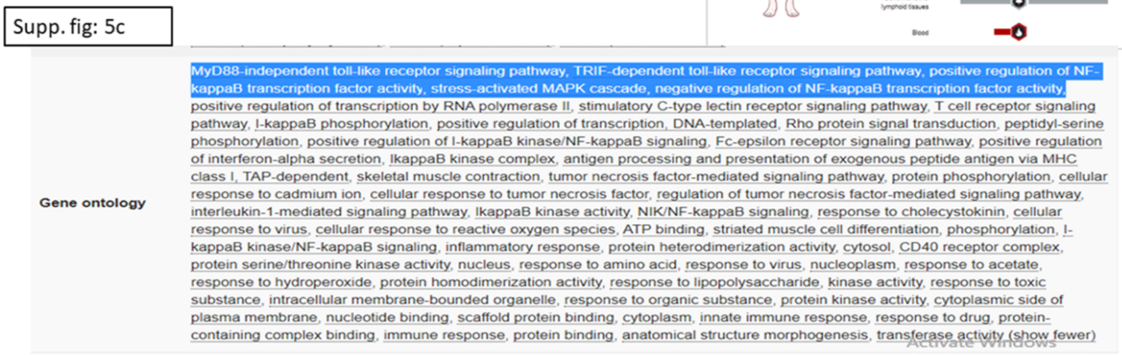

**Gene ontology**	MyD88-independent toll-like receptor signaling pathway, TRIF-dependent toll-like receptor signaling pathway, positive regulation of NF-kappaB transcription factor activity, stress-activated MAPK cascade, negative regulation of NF-kappaB transcription factor activity, positive regulation of transcription by RNA polymerase II, stimulatory C-type lectin receptor signaling pathway, T cell receptor signaling pathway, I-kappaB phosphorylation, positive regulation of transcription, DNA-templated, Rho protein signal transduction, peptidyl-serine phosphorylation, positive regulation of I-kappaB kinase/NF-kappaB signaling, Fc-epsilon receptor signaling pathway, positive regulation of interferon-alpha secretion, IkappaB kinase complex, antigen processing and presentation of exogenous peptide antigen via MHC class I, TAP-dependent, skeletal muscle contraction, tumor necrosis factor-mediated signaling pathway, protein phosphorylation, cellular response to cadmium ion, cellular response to tumor necrosis factor, regulation of tumor necrosis factor-mediated signaling pathway, interleukin-1-mediated signaling pathway, IkappaB kinase activity, NIK/NF-kappaB signaling, response to cholecystokinin, cellular response to virus, cellular response to reactive oxygen species, ATP binding, striated muscle cell differentiation, phosphorylation, I-kappaB kinase/NF-kappaB signaling, inflammatory response, protein heterodimerization activity, cytosol, CD40 receptor complex, protein serine/threonine kinase activity, nucleus, response to amino acid, response to virus, nucleoplasm, response to acetate, response to hydroperoxide, protein homodimerization activity, response to lipopolysaccharide, kinase activity, response to toxic substance, intracellular membrane-bounded organelle, response to organic substance, protein kinase activity, cytoplasmic side of plasma membrane, nucleotide binding, scaffold protein binding, cytoplasm, innate immune response, response to drug, protein-containing complex binding, immune response, protein binding, anatomical structure morphogenesis, transferase activity (show fewer)

Figure S5. Showing the retrieval of CHUK as an important intermediate in cGAS/CGAMP/STING pathway. Supp. fig.: 5a, Shows the Sting interaction between CHUK and the other genes related to TING pathway obtained from gene cards data base. Supp. fig.: 5b, shows the different tissue expression of CHUK by Human Protein Atlas data base. Supp. fig.: 5c, shows its gene ontology extracted from Expression Atlas data base.

Supp. fig: 5a

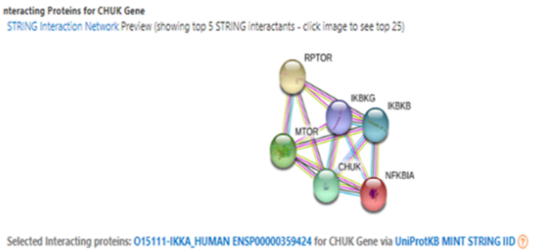

Supp. fig: 5b

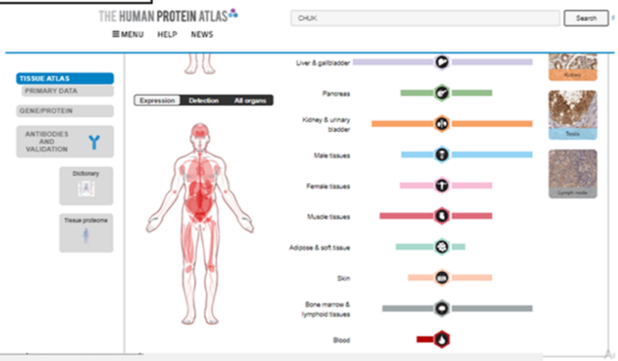

Supp. fig: 5c

Gene ontology

MyD88-independent toll-like receptor signaling pathway, TRIF-dependent toll-like receptor signaling pathway, positive regulation of NF-kappaB transcription factor activity, stress-activated MAPK cascade, negative regulation of NF-kappaB transcription factor activity, positive regulation of transcription by RNA polymerase II, stimulatory C-type lectin receptor signaling pathway, T cell receptor signaling pathway, I-kappaB phosphorylation, positive regulation of transcription, DNA-templated, Rho protein signal transduction, peptidyl-serine phosphorylation, positive regulation of I-kappaB kinase/NF-kappaB signaling, Fc-epsilon receptor signaling pathway, positive regulation of interferon-alpha secretion, IkappaB kinase complex, antigen processing and presentation of exogenous peptide antigen via MHC class I, TAP-dependent, skeletal muscle contraction, tumor necrosis factor-mediated signaling pathway, protein phosphorylation, cellular response to cadmium ion, cellular response to tumor necrosis factor, regulation of tumor necrosis factor-mediated signaling pathway, interleukin-1-mediated signaling pathway, IkappaB kinase activity, NIK/NF-kappaB signaling, response to cholecystokinin, cellular response to virus, cellular response to reactive oxygen species, ATP binding, striated muscle cell differentiation, phosphorylation, I-kappaB kinase/NF-kappaB signaling, inflammatory response, protein heterodimerization activity, cytosol, CD40 receptor complex, protein serine/threonine kinase activity, nucleus, response to amino acid, response to virus, nucleoplasm, response to acetate, response to hydroperoxide, protein homodimerization activity, response to lipopolysaccharide, kinase activity, response to toxic substance, intracellular membrane-bounded organelle, response to organic substance, protein kinase activity, cytoplasmic side of plasma membrane, nucleotide binding, scaffold protein binding, cytoplasm, innate immune response, response to drug, protein-containing complex binding, immune response, protein binding, anatomical structure morphogenesis, transferase activity (show fewer)

Activate Windows

Figure S6. Showing the retrieval of HSPA1B as an important intermediate in NOD like receptor pathway. Supp. fig.: 6a, Shows the relation between HSPA1B and NOD like receptor pathway retrieved from gene cards data base. Supp. fig.: 6b, shows the different tissue expression of HSPA1B by Human Protein Atlas data base.

Supp. fig: 7

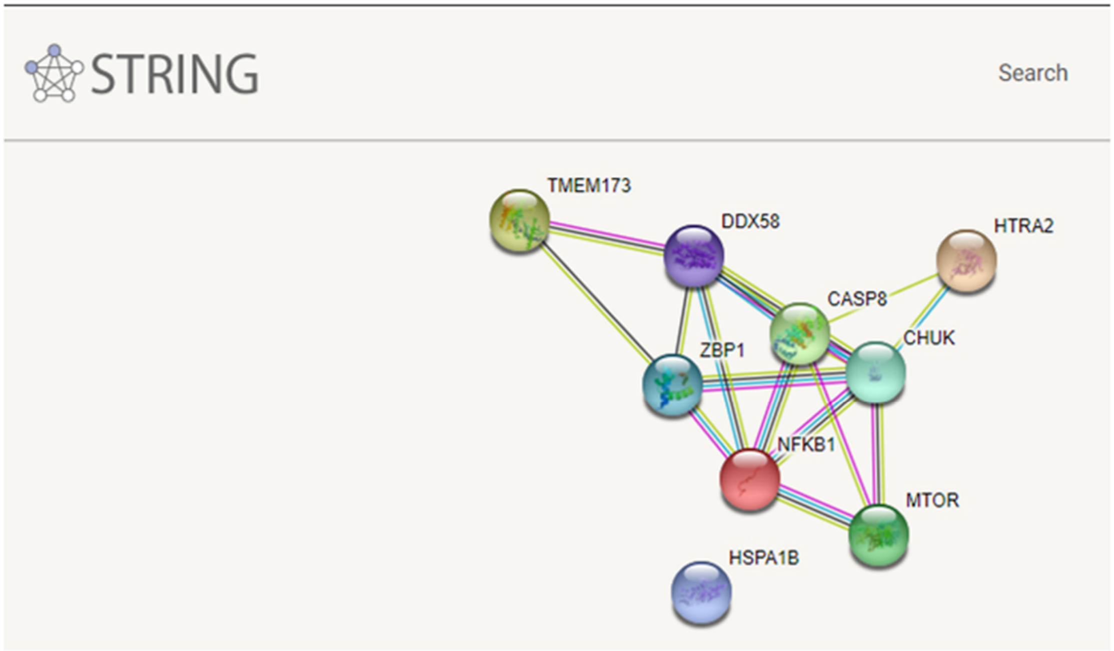

Figure S7. Showing the STRING interaction between the 6 chosen mRNAs (TMEM173, DDX58, ZBP1, CHUK, NFKB1 and HSPA1B) obtained from Gene Cards data base.
