# Supplementary material for: Identification of a Multi-Messenger RNA Signature as Type 2 Diabetes Mellitus Candidate Genes Involved in Crosstalk between Inflammation and Insulin Resistance
Source: Biomolecules. 2022 Sep 2;12(9):1230. doi: 10.3390/biom12091230 (PMC9496026; doi:10.3390/biom12091230)
Supplement: Supplementary file 1 [file biomolecules-12-01230-s001.zip › Supplementary tables.pdf]

Table S1. The research group's medical and physical characteristics.

Parameters	Healthy controls (n=45)	Pre-DM (n=44)	T2DM (n=61)	χ2	P
**Sex**				1.088	0.580 [b]
Male (53)	18 (40%)	13(29.5%)	22 (36.1%)		
Female (97)	27(60%)	31 (70.5%)	39 (63.9%)		
**Smoking**				35.637	0.000** [b]
Smoker (73)	7 (15.6%)	24 (54.5%)	42 (68.9%)		
Nonsmoker (71)	37 (82.2%)	19 (43.2%)	15 (24.6%)		
x- smoker (6)	1 (2.2%)	1(2.3%)	4 (6.6%)		
**Family history**				81.467	0.000** [b]
+ ve (78)	0 (0%)	24 (54.5%)	54 (88.5%)		
-ve (72)	45 (100%)	20 (45.5%)	7 (11.5%)		
**Parameters**	**Healthy controls**	**Pre-DM**	**T2DM**	**F**	**P**
**Age**	51.73 ± 6.9	53.27 ± 7.7	54.43 ± 7.9	**F=1.6**<br>-1.5[(a)]<br>-2.7[(b)]<br>-1.1[(c)]	**0.2** [a]<br>0.3 [a]<br>0.07 [a]<br>0.4 [a]
**FSG**	88.8±15.1	96.1 ± 17.7	194.9 ± 78.1	**F=72.1**<br>-7.3[(a)]<br>-106.1[(b)]<br>-98.8[(c)]	**0.000** [a **]<br>0.5 [a]<br>0.000 [a **]<br>0.000 [a **]
**Duration of DM**			10.59 ± 5.6	**155.4**	**0.000** [a **]
**HA1c**	3.9 ± 1.2	4.4 ± 1.4	8.1 ± 2.8	**F=64.9**<br>-0.4[(a)]<br>-4.1[(b)]<br>-3.6[(c)]	**0.000** [a **]<br>0.4 [a]<br>0.000 [a **]<br>0.000 [a **]
**HOMA-IR**	0.9 ±0.6	3.2 ± 2.5	6.1 ± 3.6	**F=48.5**<br>-2.2[(a)]<br>-5.2[(b)]<br>-2.9[(c)]	**0.000**[a**]<br>0.000 [a **]<br>0.000 [a **]<br>0.000 [a **]
**HOMA-B**	199.2 ± 20.8	154.5 ± 45.6	56.8 ± 13.0	**F=352.1**<br>44.7[(a)]<br>142.3[(b)]<br>97.6[(c)]	**0.000** [a **]<br>0.000 [a **]<br>0.000 [a **]<br>0.000 [a **]

**Diastolic blood pressure**	76.4 ± 5.1	85.8 ± 13.2	90.9 ± 10.3	**F=26.7**<br>-9.3(a)<br>-14.4(b)<br>-5.1(c)	**0.000** a **<br>0.000 a **<br>0.000 a **<br>0.01 a *
**Systolic blood pressure**	117.7 ± 8.2	130.9 ± 17.5	136.9 ± 14.5	**F=24.7**<br>-13.2(a)<br>-19.2(b)<br>-5.9(c)	**0.000** a **<br>0.000 a **<br>0.000 a **<br>0.03 a *
**BMI**	23.1 ± 4.4	33.2 ± 5.7	35.1 ± 5.4	**F=73.5**<br>-10.1(a)<br>-11.9(b)<br>-1.8(c)	**0.000** a **<br>0.000 a **<br>0.000 a **<br>0.07 a
**Cholesterol**	102.5 ± 24.9	213.9 ± 91.6	308.3 ± 61.8	**F=130.7**<br>-111.5(a)<br>-205.8 (b)<br>-94.3(c)	**0.000** a **<br>0.000 a **<br>0.000 a **<br>0.000 a **
**LDL-c**	74.7 ± 17.9	139.2 ± 52.5	213.8 ± 51.7	**F=127.4**<br>-64.5(a)<br>-139.1(b)<br>-74.6(c)	**0.000** a **<br>0.000 a **<br>0.000 a **<br>0.000 a **

a: Independent t test, b: chi square test (crosstabs test), P: P value, ** $p < 0.01$: Highly Significant, * $p < 0.05$: Significant, $p > 0.05$: Non-Significant (NS).

Table S2. Linear regression analysis for prediction of pre-diabetes.

	Unstandardized Coefficients	Standardized Coefficients	t	Sig.	95.0% Confidence Interval for B	
	B	Beta			Lower Bound	Upper Bound
BMI	0.014	0.123	1.724	0.087	-0.002	0.030
Total_Cholesterol	0.002	0.260	1.984	0.049	0.000	0.004
LDLc	0.005	0.429	3.370	0.001	0.002	0.008
zbp1	−3.857E-5	−0.051	−0.562	0.575	0.000	0.000
HSPA1B	0.001	0.118	2.486	0.014	0.000	0.002
TMEM173	5.423E-6	0.083	1.798	0.074	0.000	0.000
DDX58	8.994E-7	0.040	0.845	0.400	0.000	0.000
NFKB1	4.544E-6	0.113	2.182	0.031	0.000	0.000
CHUK	4.192E-5	0.109	1.185	0.238	0.000	0.000

CI: confidence interval, *P less than o.05 significant , BMI: body mass index, LDL-c: low density lipoprotein cholesterol.
